# Microfluidic platform combining droplets and magnetic tweezers: application to HER2 expression in cancer diagnosis

**DOI:** 10.1038/srep25540

**Published:** 2016-05-09

**Authors:** Davide Ferraro, Jérôme Champ, Bruno Teste, Marco Serra, Laurent Malaquin, Jean-Louis Viovy, Patricia de Cremoux, Stephanie Descroix

**Affiliations:** 1 Institut Curie, PSL Research University, Laboratoire Physicochimie, CNRS/UMR 168, Institut Pierre-Gilles de Gennes, MMBM group, Paris, France; 2 APHP Hôpital Saint-Louis, Molecular Oncology Unit, University Paris-Diderot, INSERM/CNRS, UMR944/7212, Paris, France; 3 LAAS-CNRS, Université de Toulouse, CNRS, Toulouse, France

## Abstract

The development of precision medicine, together with the multiplication of targeted therapies and associated molecular biomarkers, call for major progress in genetic analysis methods, allowing increased multiplexing and the implementation of more complex decision trees, without cost increase or loss of robustness. We present a platform combining droplet microfluidics and magnetic tweezers, performing RNA purification, reverse transcription and amplification in a fully automated and programmable way, in droplets of 250nL directly sampled from a microtiter-plate. This platform decreases sample consumption about 100 fold as compared to current robotized platforms and it reduces human manipulations and contamination risk. The platform’s performance was first evaluated on cell lines, showing robust operation on RNA quantities corresponding to less than one cell, and then clinically validated with a cohort of 21 breast cancer samples, for the determination of their HER2 expression status, in a blind comparison with an established routine clinical analysis.

The discoveries allowed by large-scale genomics and post-genomics have important outcomes in medicine, giving rise in particular to the development of “precision medicine”. This approach is defined by the Food and Drug Administration as the tailoring of medical treatment to the individual characteristics, needs and preferences of a patient during all stages of care, including prevention, diagnosis, treatment and follow-up^1^. To date, advances in genomic sequencing have improved the knowledge of the genetic characteristics of tumor cells and led to the identification of numerous new gene mutations or amplifications that may be appropriate for genomics-driven drug development. The panel of targeted therapies matched to specific patient’s tumor genomic alterations is fast increasing. As a consequence, the timely selection of the best therapy for each patient is becoming an increasingly difficult challenge for physicians. In parallel, important efforts are developed in clinical practice to minimize the invasiveness of diagnostic acts, leading, in particular, to a development of micro-biopsies and more generally to a reduction of the typical size of clinical samples. In order to perform assays of increasing complexity on samples of decreasing size, innovative technologies allowing fast and multigene screening analysis from minute sample material are needed^2^,^3^,^4^. We describe here such a method and validate it regarding the specific problem of HER2 (human epidermal growth factor receptor 2) status determination in breast cancer patients.

Breast cancers represent 23% of all cancers and 14% of cancer mortality worldwide. 15~20% of breast cancer patients present an amplification of HER2 and subsequent overexpression in breast cancer is associated with a more aggressive clinical behavior^5^. However, adjuvant humanized monoclonal antibody directed against HER2 (trastuzumab) or HER2-targeted tyrosine kinase inhibitors (e.g. Lapatinib) have dramatically improved survival of breast cancer patients with HER2 overexpression (HER2+), compared with patients without such overexpression (HER2-)^6^. Therefore, a reliable evaluation of HER2 expression level has become a major biomarker in daily clinical practice. The initial diagnosis of breast cancer and risk classification involves pretreatment core needle biopsies, histological diagnosis, hormone receptor and HER2 status. Recommendations for HER2 evaluation methods in breast cancers are published and periodically updated by a panel of experts to improve upon the accuracy and convenience of HER2 testing by immunohistochemistry (IHC) or Fluorescent *In Situ* Hybridization (FISH), which represent the gold standard in clinical practice^7^,^8^. Recently, we investigated the potential of Quantitative Polymerase Chain Reaction (qPCR) as an alternative to FISH, in a multicentre screening based on 840 cases, in the framework of a prospective project entitled “Support Program for Costly Diagnostic and Therapeutic Innovations” supported by the French Institute of Cancer (INCa)^9^. As a conclusion of this study, qPCR represents a reliable alternative for the quantification of HER2 expression level in cancer classification. This technique is significantly less labor-intensive and more reliable than FISH, but it remains rather expensive, notably due to the cost of reagents, and as all PCR-based diagnosis approaches, it requires rigorous procedures and environments to avoid contamination and false-positives.

A promising technique to fulfil the fast increasing demand for throughput and multiplexing associated with “precision medicine”, while reducing costs, is microfluidics. This technology allows volume reduction, automation and confinement^10^. Quake *et al*.^11^ developed a Reverse Transcription (RT) -PCR assay in microfluidic devices based on PDMS valves, an approach now commercial available in the Fluidigm IFC device. This device allows outstanding single cell sensitivity and a high throughput, thanks to the parallel processing of numerous samples versus numerous conditions (e.g. 48 × 48). This format, in which the cost per run is essentially independent of the number of samples processed, yields low cost per data point at full capacity, but is not well adapted to diagnostic screening of gene expression, in which samples arrive at the laboratory at random times, and a short time to answer is required for each sample. Droplet microfluidics, which allows compartmentalization in nanoliter volumes without complex integrated valves, has the potential to overcome this limit and, indeed, proof-of-concept experiments using droplet microfluidics were reported since then^12^,^13^. However, most droplet based approaches to RT-PCR so far, followed the same spirit as developed for “digital PCR”^14^,^15^, e.g. the generation and pooling of a large quantity of individual droplets from a single liquid sample. Higher programmability was introduced by electro-wetting on dielectric technology (EWOD)^16^ and a device performing messenger RNA (mRNA) extraction, purification and transcription has been recently described^17^. EWOD, however, requires complex microfabricated devices with electrodes, bringing back the cost issues mentioned regarding microfabricated valves^11^. It also requires an additional upstream interface for sample and reagents loading.

Here we present a new platform aimed at bringing to the clinical world the advantages of droplet microfluidics, addressing issues relevant to the specific challenges of clinical diagnosis, i.e. reliability, robustness to contamination, high multiplexing potential, versatility, user-friendliness, automation and cost reduction, while maximizing the compatibility and interoperability with currently validated and used protocols and workflows. The front-end of this platform is a simple motorized pipetting arm, which allows the direct sampling of samples and reagents from standard microtiter plates (MTP), for full compatibility with current sample storage strategies and equipment. As a main difference as compared to currently commercialized droplet platforms, such as those used in “digital PCR”, all droplets are kept equally spaced by oil in a highly confined state (a format also called “plug microfluidics” in literature)^18^,^19^. This way, inter-droplet contamination risk is reduced and the “identity” (originating well) of each droplet, built in the sequence of droplets all along the protocol, is known without need for internal tags. These differences alleviate several current limitations of droplet microfluidics^20^. The second original feature of the platform is the “magnetic tweezers” concept (Fig. 1), previously applied to ELISA^21^, and applied here for the first time to nucleic acids analysis. This magnetic tweezers approach allows the transfer of functionalized magnetic particles between subsequent droplets, bringing into the droplet microfluidics world the power of solid-state extraction and the flexibility of magnetic beads-based protocols. Here we use it to specifically capture and purify mRNA from a raw sample. Finally, the platform comprises a thermocycler, in which RT and pre-amplification PCR cycles are performed in the droplets. The whole protocol is programmed in a series of “trains” of confined droplets containing all the required reagents and samples. Several trains can be loaded sequentially and travel at constant distance from each other like in real railway. This way, multiple samples can be processed in a fully automated way in a single capillary, without any contact with external environment, reducing labour needs, risks of manipulation errors and/or contamination, in addition to a strong reduction of reagents and samples needs.

Different tests were performed in order to validate the platform, focusing on two genes of interest, the target gene HER2 and a reference gene named TBP (TATA-binding protein). As already introduced, HER2 is a major theranostic biomarker for breast cancer. TBP, a “house-keeping gene” with low variability of expression in breast cancer cells, is usually chosen in clinics as a reference in order to validate the quality of total RNA samples. A first series of experiments was performed with total RNA samples extracted from two human breast cancer cell lines: MCF7, which belongs to the luminal subgroup, has a normal expression level of HER2 and SKBR3, which presents an overexpression of this gene, displays a typical HER2+ status.

The ability of the platform to perform RT and PCR in droplets was evaluated and quantified. The performance of PCR technologies depend on many factors, including thermal control, detection, data analysis and normalization, and algorithms for Cycle threshold (Ct) determination are rather complex and apparatus-dependent. These developments are now integrated in conventional qPCR devices in a way transparent to end-users, and they constitute the frame in which biologists perform their analysis and draw their conclusions. These qPCR platforms have a relatively high sample and reagents consumption, and integrating fluorescence detection directly in the droplet format could induce strong gains regarding cost and sensitivity. However, in this first study of a new microfluidic concept, we wanted to quantify its advantages and possible disadvantages without adding in the comparison other factors, such as the sensitivity and stability of a new detection scheme, the influence of which would combine with the microfluidic part in a way difficult to unravel. Therefore, in order to allow for a more reliable comparison, we chose to perform the final steps of the analysis in a conventional qPCR machine, taking advantage of the possibility offered by the platform to collect after processing individual samples in conventional microtubes or MTP.

Then, a clinical validation of the overexpression level of HER2 in breast patient samples was performed thanks to a blind comparison versus the method previously established^9^.

## Results

### Design layout and characterization of the microfluidic platform

**Figure 1:**
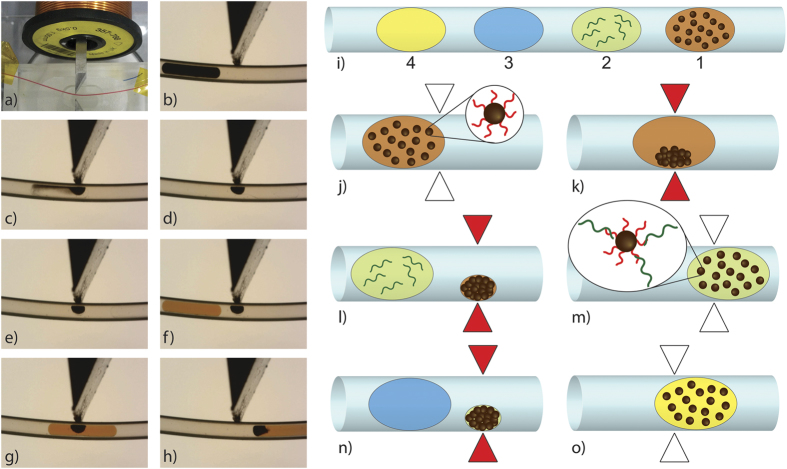
Magnetic tweezers technology and experimental workflow for RT-PCR experiment. (**a**) Picture of the magnetic tweezers with the capillary (highlighted with a red liquid). A second passive magnetic tip, placed opposite to the first with regards to the capillary, contributes to field lines shaping and to the optimization of the magnetic force^21^. (**b**–**h**) Sequence of images showing the extraction and the redispersion of magnetic beads from one droplet to another one (colored in orange), switching ON and OFF the electric current in the coil. (**i**) Scheme of a typical train of droplets used for the RT-qPCR analysis: 1) oligo-dT magnetic beads, 2) total RNA sample, 3) washing buffer and 4) RT-PCR mix. (**j**–**o**) Workflow of beads and droplets manipulation during the protocol.

The crux of the platform is the magnetic tweezers technology^21^ (Fig. 1): a soft ferromagnetic core is positioned with its body in a tunable electric coil and its sharp tip in close contact with a capillary (Fig. 1a). The capillary is filled with fluorinated oil and highly confined aqueous droplets are carried over the tip by the oil flow. Applying an electric current to the coil, a magnetic field is generated, with a peak of about 0, 35T at the tip and a strong gradient within the capillary, able to attract paramagnetic beads suspended in an initial droplet traveling in the capillary (Fig. 1c). The magnetic force is sufficient to extract the beads as a densely packed aggregate against capillary forces and retain it immobile in the oil flow. The beads aggregate is kept within an aqueous subdroplet (Fig. 1d,e), so that it is never in contact with the oil or the channel wall. When a second droplet is carried by the flow over the tip (Fig. 1f), coalescence with the magnetically trapped subdroplet spontaneously occurs. If the magnetic field is turned off, the beads are released and dispersed inside the following droplet (Fig. 1g,h) (see Supplementary Movie 1). Thanks to this, functionalized beads can be used to extract analytes of interest from a complex sample and carry them between different media, with very little carryover of the initial matrix. The procedure can be repeated using series of tweezers so that particles can be sequentially exchanged from one droplet to the other within a droplet train. For the present RT-PCR protocol, the sequence of drops is described in Fig. 1. The first droplet contains oligo-dT magnetic beads in suspension in their storage buffer. They are extracted and released in a second droplet containing the sample of total RNA (Fig. 1j–l); here mRNA is captured on the magnetic beads by hybridization (Fig. 1m). The magnetic beads are captured by a second tweezers after 5 minutes of incubation (set by the distance between tweezers and by the flow velocity), allowing the extraction of the mRNA. The cluster is then washed by a washing buffer droplet (Fig. 1n) and finally redispersed in a last droplet containing the RT-PCR mix (Fig. 1o). The whole train is then transported seamlessly to a serpentine channel in a heated element, where RT and PCR will be performed. In order to achieve full automation, a dedicated program using LabView 2011 (National Instruments) was developed to synchronize magnetic field actuation with the passage of the droplets (see Methods and Supplementary Note 1).

**Figure 2:**
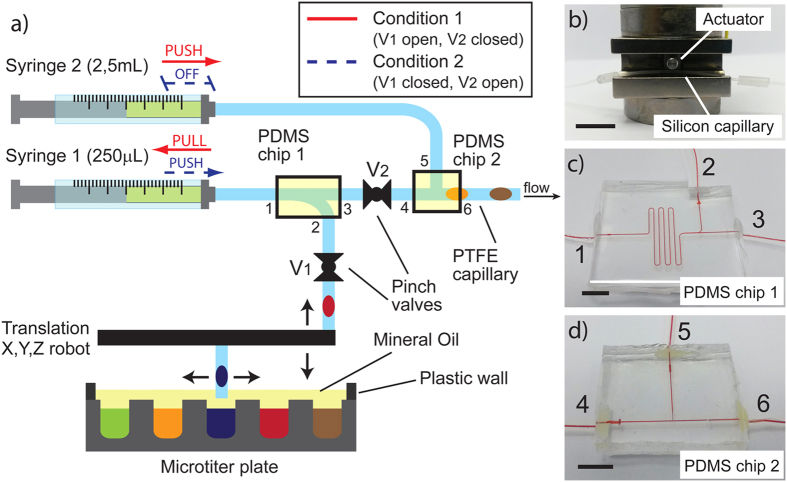
Microfluidic platform for the generation of trains of droplets. (**a**) Scheme of the droplet microfluidic generator module, which combines a MTP plate placed under a motorized pipettor arm (Cetoni), two syringes, two pinch valves (**b**) and two PDMS chips (**c**,**d**). The connections between the PDMS chips inlets and the different parts of the platform are numbered from 1 to 6 and the same numbers are reported on the scheme (**a**). Syringe 1 is connected with inlet 1 of PDMS chip 1 (Fig. 2c) while syringe 2 is connected with PDMS chip 2 (Fig. 2d) at inlet 5; inlets 2 and 3 are connected respectively to the pipettor head and to the PDMS device 2 (inlet 4), thanks to tubing passing through the two valves (V1 and V2). Finally, inlet 6 of the second PDMS chip is connected with the capillary in which droplets are driven between the magnetic tweezers. In order to generate droplets trains, while other droplets are flowing in the rest of the platform, homemade software switches alternatively between two conditions represented by red and blue lines. In condition 1, V1 is open and V2 is closed; syringe 1 is in aspiration mode generating and storing droplets in the PDMS device 1 while syringe 2 is pushing oil in the rest of the circuit. Then, in condition 2 (V1 closed, V2 open), syringe 2 is stopped and syringe 1 pushes the generated droplet train over PDMS device 2; after that, condition 1 is reactivated, and a new droplet train can be generated while the previous one is flowing in the processing system. In order to avoid pinching of a droplet by a valve, 10 mm oil spacers were introduced between droplet trains, and pipetting and valving were synchronized to ensure that valves close only onto these spacers. Each train in the series of trains can be prepared from any arbitrary combination of samples and reagents. The water and oil phase solutions are placed in the MTP, which is covered by a mineral oil film of 2–3 mm for avoiding contaminations or solution evaporations. The length of the scale bar of (**b**–**d**) is 1 cm.

Droplets trains are generated by pipetting the different solutions directly from a MTP. The use of confined droplets, which can travel over long distances keeping their initial order and spacing^22^, allows to implement in a flexible way complex protocols, built in the sequence of droplets. This leaves a lot of flexibility for programming, since the order and content of droplets can be customized at will by the end-user, including the possibility to program different protocols into successive trains. The platform, schemed in Fig. 2, is inspired from the work of Chabert *et al*.^23^, with some differences aimed at accounting for the specificities of RT and at increasing interoperability with current diagnosis platforms. The system involves a high-precision syringe pump (Syringe 1) dedicated to droplets sampling, a high volume syringe pump (Syringe 2) to push trains of droplets at constant velocity (set at 0.1 μl/s) through the device, and a compact 1-arm motorized pipettor allowing programmed sampling from any well in a MTP. Sampling is performed by sequential aspiration of sample or reagents and oil. PDMS microfluidic devices (Fig. 2c,d) are designed to operate as switching and storage junctions in order to create two independent fluidic circuits. Using those and a combination of pinch valves (Fig. 2b), the system is able to generate a train of droplets, while previously generated trains are flowing in the rest of the platform (see Methods). This approach allows a continuous operation, wherein an intermittent pipetting process generates successive trains, while previously generated trains are processed at constant speed. It also allows “on-the-flight” reprogramming, i.e. varying the samples and reagents combination for next trains, while a previously prepared train is studied, without interrupting operation.

In order to avoid any contaminations between different wells by the capillary, the same PTFE capillary was used for sampling and droplets train transport. The carrier oil was fluorinated oil (FC-40, by 3 M) containing a fluorinated surfactant (2% of 1H, 1H, 2H, 2H-perfluoro-1-decanol, by Fluorochem). In such conditions, the surface tension between the oil and the PTFE is low enough to guarantee that a small film of FC-40 is always present at the end of the capillary. Fluorinated oil also reduces the risk of molecules diffusion from the aqueous phase droplet into the continuous oil phase, avoiding cross-contamination problems^24^. This has been demonstrated performing systematically negative control tests, consisting in normal train of droplets where the RNA droplet is replaced by washing buffer, interspaced between positive trains. In particular, a negative control train was systematically included every 7 positive trains and all trains of droplets were treated using the same protocol, from the droplet generation to the qPCR amplification. In all the tests (total of more than 200 for the whole series of experiments presented in this work), the qPCR curves from the negative control droplets did not shown any amplification. An example of this can be observed in Supplementary Fig. 2, which shows raw data obtained of the amplification curves. In contrast with conventional droplet microfluidics, in which surfactants are designed to prevent droplet merging by maximizing steric repulsion between the surfactant molecules^25^, we used here perfluorodecanol, which produces stable water phase droplets in fluorinated oil and prevents wall contamination, but still allows the merging of droplets and subdroplets upon contact in a highly confined state^26^.

In order to prevent evaporation problems^27^ and to insulate the RNA samples from the external environment, reducing risks of cross-contamination and degradation by ambient RNases , a layer of mineral oil is dispensed over the MTP after having placed the solutions. In contrast with previous work^23^, mineral oil was preferred to fluorinated oil: being less dense (0.838 g/mL at 25 °C) than both water (about 1 g/mL at 25 °C) and FC-40 (1.855 g/mL at 25 °C), it stays on top of them during pipetting (Fig. 2a), without requiring the use of specific MTPs. Overall, the droplet size dispersion ranged between 1–2% for droplet volumes between 50–350 nL. During the experimental workflow droplets of 250 nL were used.

After the steps performed in the magnetic tweezers, droplets containing the purified mRNA mixed with reverse transcriptase, polymerase and primers are flown into a temperature controlled module kept at 50 °C for 5 minutes (see Supplementary Fig. 1) and the RT step is performed in the droplets. In order to evaluate the platform’s automation capability and robustness, 45 trains of droplets were continuously generated and processed without human intervention in the same experiments. They allowed analyzing two genes of interest from 7 total RNA samples and three negative controls performed in triplicate. The whole automated protocol, from droplets generation to the RT, takes about 4 hours. After that, in a first series of experiments, droplets containing the complementary DNA (cDNA) were collected in standard PCR tubes, pre-loaded with TaqMan mix, to perform the qPCR in a conventional instrument (SmartCycler, by Cepheid). In a second protocol, aimed at demonstrating the possibility to perform RT-PCR seamlessly in the platform, the droplets were subjected to a series of 15 pre-amplification by thermal cycling, before transfer to the conventional qPCR machine.

### Optimization and validation of the microfluidic platform with cell lines

#### Calibration curves for TBP and HER2

A first series of experiments was performed to check that the high surface to volume ratio intrinsic to microfluidics does not affect the efficiency of the reaction, e.g. by enzymes adsorption or loss. This was achieved by varying enzymes concentration in the RT-PCR mix (Supplementary Table 1). Increasing the concentration beyond that suggested by the supplier did not yield any decrease of Ct, whereas decrease of enzymes concentration yielded an increase in Ct only for concentrations below 20% of the standard one. This demonstrates that the concentration recommended by the supplier in macroscopic format also corresponds to a robust optimum of efficiency in our platform, so this concentration was used in following experiments.

Then, the capture efficiency of mRNA by the magnetic beads was evaluated by performing sequential capture steps from the same initial sample (see Supplementary Note 2 for details). Parallel tests performed by the droplet microfluidic platform and in conventional PCR tube, using the same protocols, show in both cases an excellent capture efficiency of 95 ± 2% for the droplet format and 93 ± 2% for the batch mode. The similarity of the efficiency in both modes demonstrates that the microfluidic format indeed exploits the good capture efficiency of the beads used here to its best extent, and does not yield any specific loss.

The purification efficiency (defined here as the rejection factor for non-messenger RNA) was evaluated by quantifying the total RNA content of the purified droplet and the waste droplet, respectively, and was shown to be better than 100X (see Supplementary Note 3 for more details).

**Figure 3:**
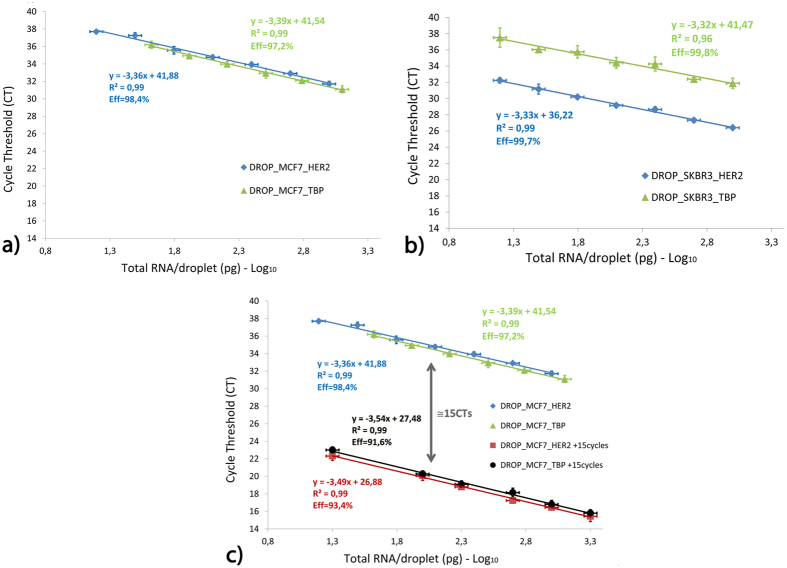
qPCR calibration curves. Calibration curves for (**a**) MCF7 and (**b**) SKBR3 cell lines for genes HER2 and TBP, represented in blue and green, respectively. Cts are plotted in logarithmic scale as a function of the total RNA concentration. The qPCR efficiency E*ff*, reported below the fit parameters, was extracted from the slope of the linear fits. (**c**) Calibration curves for MCF7 without (blue and green points and fits) and with (red and black points and fits) 15 PCR pre-amplifications cycles: as expected, the curves are shifted of about 15 Cts. Error bars are not visible when they are smaller than the point.

Calibration curves for TBP and HER2 genes were then prepared with total RNA quantities ranging from 1.5 ng/drop to 15 pg/drop, corresponding to 6 ng/μl to 60 pg/μl; this range is lower than the typical concentrations used for the routine quality check for clinical samples (6 and 25 ng per assay). The lowest calibration point, about 15 pg, roughly corresponds to the total mRNA content of a single cell or, considering the typical expression level of HER2 in breast cancer lines, between 10 and 1000 copies of target mRNA per droplet. Figure 3a,b show the results obtained for MCF7 and SKBR3 cell lines, respectively. Each curve is the average of a triplicate of the same experiment repeated three times, starting from the same MTP. An example of raw data obtained is provided in Supplementary Fig. 2. The Cts horizontal error bars for the RNA/droplet concentrations are evaluated by the propagation of error on the droplet volume, while the verticals error bars are given by the standard deviation of the triplicate (CV < 1%). Thus, the error bars in Fig. 3 represent the variations due to our new setup and protocol, avoiding possible external sources of error such as degradation of the RNA sample or inaccuracies of MTP preparation. This point will be fundamental for the clinical validation, in which a sample calibrator has to be analyzed at the same time and with the same protocol as the patient samples. Calibration curves in Fig. 3 are linear in the investigated range (R^2^ > 0.96), demonstrating that the efficiency of mRNA capture and of the RT reaction performed in droplets are not influenced by the initial quantity of mRNA. The efficiencies (E*ff*) of the qPCR, derived by the curves’ slope reported in the graphs, are always higher than 97%. As expected, the Cts obtained for TBP do not differ significantly between the two cell lines. For HER2, in contrast, SKBR3 shows Cts about 5units smaller than those of MCF7, in line with the HER2+ and HER2- status of these cell lines.

It is also worth noting that these results confirm the absence of contamination between droplets, already demonstrated by negative controls presented in the first part of the results section, above. All data were derived from different trains of droplets performed in the same capillary, and comprising initial RNA concentrations over two orders of magnitude of concentration. In particular, in some cases, the train at lowest concentration are generated directly followed a train at the highest concentration. If some cross-contamination occurred the measured Ct should have a larger dispersion at low RNA concentrations than at high, disrupting the linearity of the calibration curves.

Finally, the possibility to perform RT-qPCR in a single step in the droplet was evaluated by an additional series of experiments performed on total RNA from MCF7 in which, after RT, the droplets trains are arrested in the temperature unit, and subject to PCR. Ct values in PCR depend to some extent on the apparatus and sensitivity of the detection so, in order to allow for a more quantitative validation, we chose a hybrid method in which 15 temperature cycles are performed in the capillary and the droplets are then transferred to the SmartCycler for final qPCR analysis. The results (Fig. 3c) show that, within experimental error, the calibration curves obtained are shifted by 15 Cts as compared to the previous ones, indicating that the efficiency of the PCR is similar in the droplets and in the conventional thermocycler. Moreover, the slopes of the graphs with and without 15 cycles of pre-amplification are also comparable, suggesting that no additional loss or degradation of enzymes occurred during thermal cycling in the droplet, and that the in-drop amplification provided DNA fragments of a similar quality as those obtained in the SmartCycler. Combined together, these results demonstrate that RT-qPCR can be performed in the highly confined droplets of the platform, without loss of reproducibility, quality and performance and without inter-trains contamination.

### Clinical Validation with breast cancer patient samples

**Figure 4:**
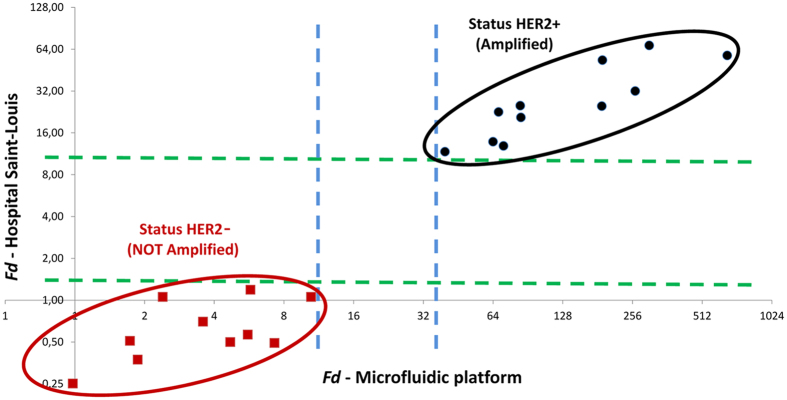
Correlation between hospital and microfluidic platform results. Graph in Logarithm scale (base 2) of the 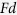 obtained by the Saint-Louis Hospital platform versus the 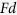 obtained by the droplet microfluidic platform. Samples disclosed *a posteriori* as HER2- samples are plotted in red, and HER2+ ones in black. The dashed lines help to identify the excluded zone between the two populations: blue for the droplet platform (10.48–39.67), green for the hospital (1.5–11.71).

Clinical validation was performed in a blind protocol, using 21 samples of total RNA extracted from percutaneous biopsies of breast cancer patients of Saint-Louis Hospital in Paris (France). The samples were analyzed, independently and in parallel, by classical RT-qPCR at the hospital (see Methods) and by the droplet microfluidics platform, using the SmartCycler as end-line quantitative analyzer, as described above for cell lines. The total RNA quantities used for this validation were, respectively, 20 ng per test for the Hospital, and 250 pg per test (or 250 pg/droplet) for the microfluidic platform. All results were analyzed with the 2^−ΔΔCt^ Method^28^,^29^, which is well established for RT-qPCR. This strategy allows the normalization of the expression of the gene of clinical interest (HER2) to a reference having a stable expression level between individuals (in our case TBP) and also with a reference sample without HER2 overexpression (MCF7 cell lines for the droplet platform and a pre-defined set of samples for the hospital platform). This allows to take into account possible differences of RNA or DNA quality and/or amplification between the two genes. The result of this analysis is the Fold difference *Fd*, which quantifies the difference in expression between the patient sample and the control sample: ideally, *Fd* ≈ 1 would mean no overexpression and the higher is *Fd*, the higher is the expression of the gene. In practice, however, there is no absolute reference of what is a “normal” expression, both for HER2 and TBP, and measured expression levels depend to some extent on the details of the experimental procedure, so the *Fd* threshold of positivity has to be evaluated on a heuristic basis, for a given platform. In the diagnostic laboratory at Saint-Louis Hospital, the 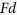
cut-off ratio to consider a sample HER2 negative or positive was determined on a tumor training set using univariate partition method (XLSTAT software) and correlation with IHC–HER2 expression. Final result were expressed as a normalized ratio considered as over-expressed if *Fd* >7^9^,^30^. The two sets of data with the Ct and *Fd* values are reported in the blue and yellow columns of Supplementary Table 2. *Fd* values obtained on the droplet platform are systematically higher than those obtained at the hospital. Such a discrepancy could be expected considering the relative nature of this parameter and our use of a different reference in the two environments. This complication can be overcome by a correlation analysis, as previously proposed^31^. The linear correlation between the two sets of data yields a Pearson Correlation Coefficient 0.84, showing good correlation. Most importantly regarding diagnosis, the correlation graph (Fig. 4) between the droplet and hospital platforms shows an identical segregation of positive and negative patients, plotted in red and black, respectively. By definition, data from the hospital segregate in two data sets on each side of the 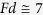
threshold. More specifically, in this cohort, the highest *Fd* of negative patients is at 1.19 and the lowest positive is at 11.71 (green dash line). All data obtained on the microfluidic platform segregate together with the data from the hospital, the highest negative being at 10.48, and the lowest positive at 39.67 (blue dashed lines).

## Discussion and Conclusions

We presented a novel droplet microfluidics platform for gene expression quantification by RT-qPCR. As already introduced above, the progress of “precision medicine”, in which an increasing number of molecular biomarkers must be tested on the same patient sample, is limited by conventional techniques, in which large quantities of samples and reagents are requested for every single assay. Additionally, gene expression information is carried by mRNA, which only represents 1–3% of the total RNA and is a more fragile and delicate nucleic acid than DNA. Finally, even minimal contamination by post-PCR amplification products from other reactions could easily yield false positives. To overcome these difficulties, very strict, time consuming and space-intensive workflows are required. These requirements, together with the cost of reagents, constitute a hindrance to the spreading of precision medicine approaches in less-favored settings and a cost burden on the health system in more favored countries.

The microfluidic platform developed here provides a path overcoming these limitations, while remaining compatible with the standard equipment and sample storage available in diagnostic laboratories. Starting from total RNA samples distributed in conventional microtiter plates, this droplet platform permits the generation of different trains of droplets allowing mRNA extraction by magnetic beads, washing, redispersion in RT-PCR mix and finally the thermal steps for RT or RT plus pre-amplification cycles of PCR, all in droplet format. Various combinations of samples and RT-PCR mixes can be programmed dynamically from the same set of samples and reagents.

The platform was first validated using total RNA extracted from MCF7 and SKBR3 cell lines. Calibration curves for both TBP and HER2 genes expression levels were found linear between 1, 5 ng and 15 pg of total RNA per droplet. As mentioned above, it is worth noting that the smallest concentration of total RNA used in this series is comparable with the RNA concentration of a single cell (about 20 pg). Therefore, although not relevant to the present clinical application and validation, our platform is suitable to perform RT-qPCR from single cells. Since technologies for single cell encapsulation in droplet exist^13^,^32^, this could open the route to a strong simplification of single cell expression studies.

During these tests, as expected, MCF7 and SKBR3 cell lines showed normal and overexpressed HER2 gene level, respectively, and the same level of TBP expression. Additionally, the curves were found parallel, yielding PCR efficiencies between 97–99%, allowing their relative expression analysis. When 15 cycles of PCR pre-amplification are performed in the droplet following RT, a shift of 15 cycles in the calibration curves is obtained, showing that the platform allows inline mRNA purification, reverse transcription and DNA amplification with an efficiency comparable to that observed in conventional PCR machines. To date, in the routine analysis developed at Saint-Louis Hospital, total RNA is used to minimize manipulations and risks of contamination. Indeed, mRNA purification is not mandatory for all applications and is often avoided in conventional protocols. In our platform, however, this step can be included without additional sample manipulation or risk of contamination, since it is performed entirely in the microfluidic capillary. Implementing this additional purification is thus a way to increase the robustness of the method, by getting rid of a large majority of spurious RNA, at no additional risk and with negligible increase in the whole process time. Additionally, it is interesting to note that new technologies such as RNA sequencing, require highly purified samples to perform multiplex reactions and tend to use more and more often additional magnetic beads-based purification steps to avoid any non-specific reactions^33^. Therefore, we believe that our platform, performing in “native” mode mRNA extraction by magnetic beads, Reverse Transcription and PCR pre-amplification, will also make it an interesting front-end technique for these new massively parallel technologies^34^,^35^.

A blind test versus an established routine clinical diagnosis was then performed on a cohort of 21 patients for the determination of HER2 amplification status, using the 2^−ΔΔCt^ Method, typical for qPCR analysis. The results show a good quantitative correlation of 0.84 between the *Fd* values. Moreover, in Fig. 4 the two populations of normal and overexpressed HER2 tumors are well identified, demonstrating that all the 21 patient samples studied were well characterized by the microfluidic platform in the blind test.

The platform used here allowed the processing of one sample every 3 minutes, i.e. about 500 samples per day. This is quite sufficient for most clinical diagnosis expression needs. Indeed, in research, experiments can be pooled and synchronized, in order to take advantage of devices processing in parallel many samples, but in clinical diagnosis, the ability to provide a fast response for each patient at a low cost is often as important or more important than theoretical throughput. Strategies involving parallel processing in disposable chips are thus poorly adapted, since the operation cost per chip is essentially independent of the number of samples processed in the chip, so that samples must be pooled up the full capacity of the chip or close to it, before processing to keep costs low. Our system, in contrast, does not use disposable chips, samples are processed sequentially and reagents are well protected under oil in the machine and can be kept for re-use on-demand. Therefore, the typical one hour response time can be maintained without significant cost increase from a single sample per day to the maximum rate of 500 samples per day, making the platform particularly suitable and cost-efficient for situations in which the number of samples to treat per day can vary widely, typically from a few to several hundred. It should be noted, however, that in order to best fulfill clinical requirements, the machine used here was aimed at flexibility but not optimized for speed. For higher throughput applications, the use of a more industrial pipetting stage, for instance, could allow the parallelization of the droplet generation step. Additionally, both valves and magnetic tweezers used here could accommodate more capillaries with little modification, so the throughput of the system could be increased by one or two order of magnitudes without major modifications.

The presented microfluidic platform shares with previous droplet microfluidic systems the use of oil to compartmentalize aqueous media in multiple nanoliter scale microreactors and thus, the possibility to considerably reduce reagents and sample consumption but, besides this, it has very different aims. Current droplet microfluidic systems use high-throughput generation of droplets from one or a few streams, to achieve thousands to millions of independent reactions in discretized samples of the same mix. This has opened, for instance, the route to digital “one molecule per droplet” approaches, leading to spectacular increases in sensitivity in PCR^14^, but the cost of analysis per sample remains high. Here, in contrast, we use compartmentalization to increase automation and reduce volume, but remain in the “one reaction per sample” paradigm. In other words, a combination of a single droplet of sample and a single droplet of PCR mix is sufficient for typing the sample regarding to the corresponding sequence. As consequence, sample and reagents consumptions are directly commensurate with the volume ratio between a single droplet and a MTP well. This, combined with the magnetic tweezers permitting droplet-to-droplet extraction, purification and transfer, allows the implementation of essentially any protocol currently implemented in pipetting robots. For instance, in this study, starting from the same liquid samples, we easily introduced in our protocols triplicates assays and negative control tests, which are fundamental in diagnostic analyses. The microfluidic platform also shares with current robotized platforms MTPs as common entry and exit points, allowing easy evolution from existing workflows. However, it presents several advantages as compared to conventional robots, which remain in the MTP format through the whole protocol; at first, the reduction of the sample volume and RT reagents needed to obtain a data point is reduced by a factor between 40 and 200. Even smaller volumes could be sampled with syringes of smaller diameters, but we did not consider this useful here, since a X40 volume reduction, in combination with the absence of microfabricated disposables, and a strong reduction in plastic consumables, are already sufficient to decrease consumables cost to a level negligible as compared to other costs in the workflow, such as blood sampling and labor. Additionally, any sample contained in a single well (typically 20 to 100 μl) can be typed versus up to 100 PCR mixes contained in different wells or, alternately, a single PCR mix of 20 to 100 μl can be used to screen up to 100 samples, in a fully programmable manner and in any combination of samples versus mixes. This opens the route to multi-biomarkers screening from the same sample volume, to costs reductions and to the implementation of diagnostic sequences based on decision trees, without additional manipulation; for instance, a new train of droplets can be prepared from the same sample well with different primers, following the result of a first test, in an operator-decided or automated way. Another advantage is a reduction of risks of human error or contamination, either by RNase (sample degradation) or by DNA (false positives), thanks to the fact that operations are performed under oil all along the protocol. Indeed, the droplet platform was installed in a multi-purpose biochemistry lab, in which various other experiments involving amplification of human DNA were also performed, without pre-PCR and post-PCR segregation, and no event suggestive of contamination was observed during the whole study. This contamination control advantage works both ways, i.e. it can also increase the protection of users, for the manipulation of potentially infectious samples.

Finally, the microfluidic platform is very versatile regarding protocol development: it can implement in a fully automated manner most existing protocols involving magnetic beads and use existing kits, except for a widely reduced reagents consumption, having an important impact on reagents cost. In the experiments presented here, this advantage is somewhat reduced by the final step, performed in a conventional thermocycler for the sake of clinical comparison. This step still requires the same quantity of polymerases and primers than in conventional methods. However, we have demonstrated that RT and qPCR can be performed in the same droplet. This opens the route to the development of a next generation device, comprising on-chip fluorescent detection, as already demonstrated in several proof-of concept experiments^36^,^37^,^38^. This improvement has the potential to further increase sensitivity, since it will avoid the dilution performed during droplet collection, in the present protocol, and also reduce the consumption of PCR reagents. Thus, either in the form presented here, as a front-end device for the preparation of direct or pre-amplified cDNA, or as a fully integrated device including detection, we believe that the droplet microfluidics and magnetic tweezers technology presented here will bring considerable gain in cost, robustness and simplicity of operation for a multiplicity of nucleic acid analysis applications, in particular in diagnosis.

## Methods

### Droplet microfluidic platform description:

The flow control part of the platform is designed to generate a train of droplets by sequential pipetting, while other droplets already generated are processed at constant and controlled speed in the rest of the platform. The platform comprises two syringe pumps (Nemesys, by Cetoni GmbH) equipped with 250 μL and 2, 5 mL syringes (by SGE), a motorized pipettor arm (Rotaxys, by Cetoni GmbH), two pinch valves (by NResearch Inc.) that are alternatively open and closed. Two PDMS devices are used for capillary connections (see Fig. 2). A 384-wells MTP is used for reagents and samples storage. A volume of 25 μL of each solution is distributed in individual wells placed below the motorized pipetting arm. Mineral light oil (by Sigma-Aldrich) is then spread on the plate in order to avoid reagents evaporation. The MTP is placed on a home-made holder that can be moved in X, Y, Z directions by a manual stage (Thorlabs). Once the pipetting capillary is aligned with the reference well, software controlling the motion of the pipettor’s arm and the syringe is operated. Capillary is moved from one well to the other, pipetting in each one the desired volume of liquid and inserting between each water droplet a fluorinated oil plug. Considering the volume capacity of a single well and the volume of the droplet and oil phases, it is possible to generate from a single solution of 25 μl about 100 droplets of 250 nl.

Figure 2 schematically describes how the different parts of the platform are connected and how the platform allows droplet generation while the previous trains are flowing in the processing system. The pipetting takes about 1 second per droplet. Including the time needed for arm motion and oil pipetting, one train of droplets can be generated and manipulated between the valves and the PDMS devices in about 2–3 minutes. The PDMS connectors were prepared by standard double replica molding techniques, starting from a brass mold made by micro milling (see Supplementary Fig. 3). The pinch valves used operate by clamping an elastic capillary by a tip controlled by an electromagnet (see Fig. 2b). PTFE capillaries used for droplet manipulation cannot be used for this purpose due to their poor elastic proprieties. Therefore, in the valves they were replaced by a silicone capillary having the same internal size than the PTFE one (0.31/0.64 mm ID/OD, by Helix Medical). The connection between the two different capillaries was achieved using a third silicon capillary (ID = 0.50 mm, OD = 1.3 mm, by Deutsch & Neumann). To ensure good wettability by fluorinated oil, PDMS devices and silicone capillaries were treated with a solution of (Tridecafluoro-1, 1, 2, 2-tetrahydrooctyl)trichlorosilane in FC-40 (5% w/w) for 1 h.

When leaving PDMS connector 2, droplets are driven in a single seamless capillary between i) the two magnetic tweezers made of two magnetic coils (copper wire of 71 cm and 1 mm diameter, by Radiospares) surrounding soft ferromagnetic tips (AFK502 alloy, by Aperam) and ii) the thermocycler provided by Prime Techne and further adapted. Two Macro Objective (MLH-10X) mounted on two low cost CMOS Cameras (acA1300–60 gm, by Basler) and white LED back light illumination (by Schott Lighting and Imaging) were used for droplet observation and image analysis for magnetic tweezers control (see Supplementary Note 1). The cameras are connected to the computer by the NI PCIe-8236 card (National Instruments) and the communication between the home made LabVIEW software and the magnetic coils is achieved by the NI USB-6525 interface (National Instrument). Since the droplet generator and the magnetic tweezers modules can work at the same time, after preparation of the samples and reagents MTP, any combination of samples and reagents can be processed in any order and numbers of iterations, until exhaustion of the volume (about 500 analyses per sample for an initial load of 100 μl).

### Samples

#### Cell lines

Human breast cancer cell lines SKBR3 (HER2-like phenotype) and MCF7 (luminal phenotype) were acquired from ATCC. They were cultured in DMEM culture medium supplemented with 10% FBS and 2% glutamine without antibiotics. They were harvested every week to amplify enough material for one year use of controls.

#### Patient cohort samples

The retrospective study was conducted on 21 breast cancer pre-treatment core-needle biopsies. Paraffin-embedded sections of AFA-fixed biopsies, used for the histological diagnosis were assessed by immunohistochemistry (for estrogen receptor ER), progesterone receptor (PR) and HER2 content. Tumours were considered “HER2-positive” if more than 30% of cells showed definite membrane staining. Control by FISH or SISH or qPCR was done for ambiguous cases. Frozen samples were used for the present study. They were processed under RNAse-free conditions. They represent a part of a more extensive protocol analysing diagnosis markers (ER, PR and HER2) and proliferation markers. The protocol and the ancillary studies were reviewed and approved by an ethics committee (CPP Ile de France IV, France, n°2013/27NICB, 30/07/2013).

### Total RNA extraction

Cell lines and tumors’ nucleic acids were extracted by phenol/chloroform procedure^39^ and qualified and quantified using Bioanalyzer (by Agilent Technologies) and Nanodrop 2000 instruments, respectively. Tumor cell purity and presence of *in situ* carcinoma were assessed on adjacent tumor H&E-stained sections, by the referent pathologist^30^.

### 2^−ΔΔCt^ Method for folding difference (*Fd*) evaluation

The 2^−ΔΔCt^ Method^28^,^29^ was used for the evaluation of the folding difference values starting from the Cts obtained by the droplets platform and the conventional hospital protocols. It proceeds as follows: at first, the Δ*Ct* is defined as the difference in Ct between the gene of interest (HER2) and the reference gene (TBP), both for the sample and for the reference:


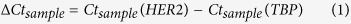



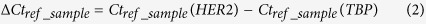


where “*ref_sample*” represents the normal control sample. Then, ΔΔ*Ct* is calculated as the difference between the two Δ*Ct*:


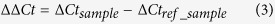


finally, the Fold difference (*Fd*) value is calculated with the following equation:


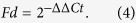


### Quantification of HER2 overexpression RT-qPCR – hospital protocol

First-strand cDNA synthesis was performed with 1 μg total RNA using Superscript II Reverse Transcriptase (Invitrogen Corporation) in a final volume of 25 μL. Quantitative PCR were performed on LightCycler 2.1 instrument (Roche Diagnostics). HER2 overexpression was evaluated by relative quantification using TATA-binding protein as endogen control (TBP) using 20 ng cDNA as previously described^30^. A 5 μL diluted sample of cDNA (20 ng) was added to 20 μL of the PCR mix. The thermal cycling conditions comprised an initial denaturation step at 95 °C for 10 min, 45 cycles at 95 °C for 15 sec, and annealing temperature, 60 °C for 1 min.

Final result were expressed as a normalized ratio *Fd* considered as over-expressed if *Fd* > 7. The cut-off ratio was determined on a tumors training set using univariate partition method (XLSTAT software) and correlation with IHC–HER2 expression as previously published by our group^30^.

### Preparation of the solutions for RT-qPCR performed in the droplet platform

Before transferring into micro titer plate, total RNA, stored at −80 °C, was quantified & qualified using Nanodrop&BioAnalyzer (Agilent) instruments. Desired total RNA quantities were diluted into Binding Buffer provided with the DynabeadsOligo(dT)25 Kit (61002, Life Technologies) and BSA (B14, Thermo Scientific) was added to a final concentration of 0.4%. For the preparation of the paramagnetic beads solutions for droplet generation, after resuspending Dynabeads in the vial, 100 μL was transfer to a tube and placed in a magnet for 1 min. The supernatant was discarded and Dynabeads were resuspended into 100 μL of Binding Buffer, well mixed and placed in a magnet for 1 min. The supernatant was discarded and Dynabeads were resuspended into a 25 μL mix composed of 23.5 μL Binding Buffer, 0.5 μL 20% BSA, 1 μL SUPERase In™ RNase Inhibitor (AM2694, Life Technologies). Two 1.5 ml tubes containing a 50 μL Washing Mix (48 μL Wash Solution, 1 μL 20% BSA, 1 μL RNase inhibitor) were prepared. CellsDirect™ One-Step qRT-PCR kits (11732, Life Technologies) was used to prepare the Reverse transcription mix as follow: 12.5 μL of 2X Reaction Mix, 1 μL of anchored Oligo(dT)23VN (S1327S, NEB), 1 μL of each Forward and Reverse primers (custom primers to study HER2 or TBP or Actin b from IDT), 1 μL of SuperScript® III RT/Platinum® Taq Mix with RNaseOUT™ Ribonuclease Inhibitor and qsp to 25 μL with DEPC-treated water. Each solution was well mixed by pipetting before transferred into 384-well titer Plate.

After the microfluidic process (mRNA capture, washing, Reverse transcription), droplets containing cDNA were recovered into 1.5 mL tubes with 5 μL of DEPC-treated water. qPCR reactions were performed in the SmartCycler instrument (by Cepheid), with a mix composed of: 12.5 μL of 2X Reaction Mix, 1 μL of each Forward and Reverse primers (custom primers to study HER2 or TBP or Actin b from IDT), 0.5 μL of TaqMan™ Probe (custom probes to study HER2 or TBP or Actin b from IDT) 1 μL of SuperScript® III RT/Platinum® Taq Mix with RNaseOUT™ Ribonuclease Inhibitor, 5 μL of cDNA output and qsp to 25 μL with DEPC-treated water. The thermocycling program was: an enzyme activation step at 95 °C for 120 sec, followed by 50 cycles of 95 °C/15 sec and 60 °C/30 sec.

## Additional Information

**How to cite this article**: Ferraro, D. *et al*. Microfluidic platform combining droplets and magnetic tweezers: application to HER2 expression in cancer diagnosis. *Sci. Rep.* **6**, 25540; doi: 10.1038/srep25540 (2016).

## Supplementary Material

Supplementary Information

Supplementary Movie 1
