# Supplementary material for: Microfluidic platform combining droplets and magnetic tweezers: application to HER2 expression in cancer diagnosis
Source: Sci Rep. 2016 May 9;6:25540. doi: 10.1038/srep25540 (PMC4860594; doi:10.1038/srep25540)
Supplement: Supplementary Information [file srep25540-s1.doc]

**SUPPORTING INFORMATION**

**Microfluidic platform combining droplets and magnetic tweezers: application to HER2 expression in cancer diagnosis**

Davide Ferraro^1^, Jérôme Champ^2^, Bruno Teste^1^, Marco Serra^1^, Laurent Malaquin^3^, Jean-Louis Viovy^1^, Patricia de Cremoux^2,*^, Stephanie Descroix^1,*^

^1^ Institut Curie, PSL Research University, Laboratoire Physicochimie, CNRS/UMR 168, Institut Pierre-Gilles de Gennes, MMBM group, Paris, France.

^2^ APHP Hôpital Saint-Louis, Molecular Oncology Unit, University Paris-Diderot, INSERM/CNRS, UMR944/7212, Paris, France.

^3^ CNRS, LAAS, Toulouse, France.

^*^ Corresponding authors: Stephanie Descroix (stephanie.descroix@curie.fr) and Patricia de Cremoux (patricia.de-cremoux@aphp.fr)

**Supplementary Movie 1:**

**Illustration of the extraction/redispersion process through magnetic tweezers.** Two confined droplets pass sequentially in the capillary: the first contains magnetic beads and the second colored water. When the droplet is passing close to the magnetic tip, the magnetic field is turned ON, by application of an electric current to the coil. Magnetic beads are trapped in a cluster and further extracted. When the following droplet is passing, the magnetic field is turned OFF and beads are released in the droplet. Magnetic tweezers and capillary filled with red color solution are shown in the inset of the movie.

**Supplementary Note 1:**

**LabVIEW program for magnetic tweezers control:** Two cameras acquire real-time images of the capillary next to the tweezers. In order to reduce processing complexity, Regions of Interest (ROIs) corresponding to the zone close to the magnetic tips are defined and all the processing is performed in these ROIs (see Supplementary Fig. N1).

The setup automation allows the control of magnetic tweezers operation during the extraction of magnetic beads from a droplet and their redispersion in a different one. The activation of the magnetic coil (ON/OFF) is triggered by a software analyzing a ROI in the vicinity of the coil tip, which recognizes the arrival of the droplet (meniscus) and the presence of magnetic beads (light intensity in the droplet). This is performed through an automated image acquisition and processing step.

The program was developed using National Instruments LabView 2011 software. The analysis workflow is the following: i) detection of magnetic beads inside a droplet and triggering the activation of magnetic tweezers in order to capture them; ii) identification of the meniscus of the target droplet in which beads have to be released: this detection triggers the turning OFF of the magnetic tweezers;


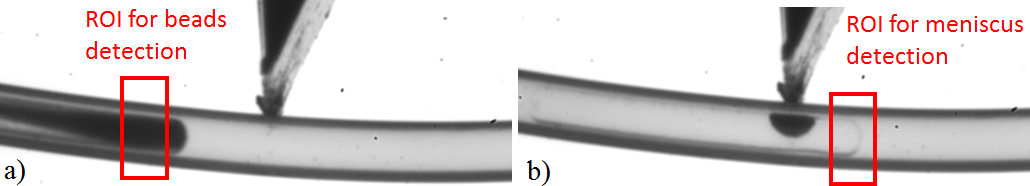


**Supplementary Figure N1:** Definition of Region of Interest (ROI) for a) beads and b) meniscus detection. Observing the two ROIs is possible to notice that the contrast is high in the case of the beads detection while is weak for the meniscus detection. In this second case, inline processing has been implemented for meniscus identification.

The core of the detection step is the computation of the normalized cross-correlation between the digital images captured by the camera and a template digital image of the object to recognize. The detection step is based on a pattern matching method, already implemented in the NI Vision module of LabView 2011. This algorithm consists in two stages: learning, performed by the package “IMAQ Learn Pattern 2.vi” and matching, based on “IMAQ Match Pattern 3.vi”. During the second step the program associates a high or low score in case of a good or a bad matching result, respectively. In the case of the detection of beads inside a droplet, the image contrast is good enough to allow identification. Due to the poor contrast provided by the oil/water interface (see Fig. N1b), inline image processing steps were performed during the acquisition to detect the meniscus position. This involves a background subtraction, which is not computationally expensive. The background subtraction (IMAQ Subtract VI) is calculated as the intensity difference between the current frame image in the real-time acquisition (see Supplementary Fig. N2a) and a static background image (see Supplementary Fig. N2b). The resulting difference image shows a better contrast but also a lot of random noise mainly due to the camera CMOS sensor (see Supplementary Fig.N2c). Therefore, an additional median filter (IMAQ Nth Order VI*)* was applied to reduce this noise (see Supplementary Fig. N2d). Finally, a thresholding (*IMAQ Threshold.vi)* generates a binary image (see Supplementary Fig. N2e). This last image is compared in real time to a reference image, as described above for beads detection.


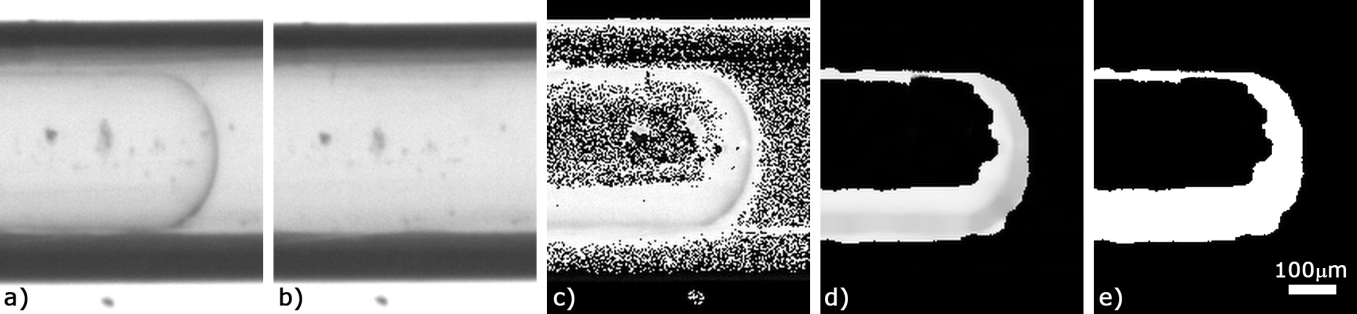


**Supplementary Figure N2:** Example of the inline real time image processing: a) ROI for meniscus detection of the real-time frame; b) background image; c) result of the background subtraction on real-time acquisition, small spots are due to random noise of the camera; d) median filter results for noise reduction; e) binary image obtained by thresholding process.

According to the strategy described in the paper, the first tweezers of the platform must release the captured beads in the following droplet containing the total RNA sample. Then, after the beads capture and the mRNA hybridization, a second set of tweezers retains the beads during the passage of a washing buffer droplet and finally release them in the RT-PCR mix droplet. To apply this protocol, each droplet should be counted. We define the result of the detection of a meniscus in a real-time acquired frame as '1' if a meniscus is recognized and ‘0’ otherwise. The presence of a '0 🡪1' transition identifies the presence of a meniscus. To reduce computation time, only two consecutive results of the detection are stored in two variables that are continuously updated when a new frame is acquired. Therefore, a meniscus is detected when the value of the variable associated to the current image is ‘1’ and the one containing the result of the previous frame is ‘0’. This detection triggers the increment of a counter variable, initialized at zero and containing information on the number of detected meniscus, used for triggering the redispersion of beads in the adequate droplet. After this step, counter is re-initialized to process the following train of droplets.

**Supplementary Note 2:**

A specific experiment, screening the TBP housekeeping gene in total human RNA (by Agilent Technologies), was achieved to evaluate the capture efficiency of the microfluidic platform. In detail, the experiment started as in the diagnosis protocol: after the first mRNA extraction, a second pool of magnetic beads, coming from another droplet, are redispersed in the total RNA droplet solution. As for the standard protocol, after the same incubation time, these magnetic beads are captured again by the magnetic tweezers, passed through the washing buffer and finally redispersed in the second RT-PCR mix droplet. This protocol was achieved thanks to the possibility of moving the droplets train forwards and backwards in the capillary by the syringe pump. After that, as for the conventional protocol, both droplets containing magnetic beads dispersed in the RT-PCR mix are treated by the thermocycler for reverse transcription, and finally collected in qPCR tube for final analysis by the SmartCycler. Two different Ct values were obtained from the first (Ct_1_) and the second (Ct_2_) extraction, respectively. To better evaluate the capture efficiency, triplicate experiments were systematically performed and the same protocol was implemented in conventional 200μL tube. An example of the raw data is plotted in Supplementary Fig. N3. The averaged results, reported in Supplementary Table N1 show that for both droplet and tube experiments, there is a difference ∆Ct of about 4 Cts between the first and the second mRNA capture, showing that the capture efficiency is limited mainly by the magnetic beads and not by the microfluidic format.


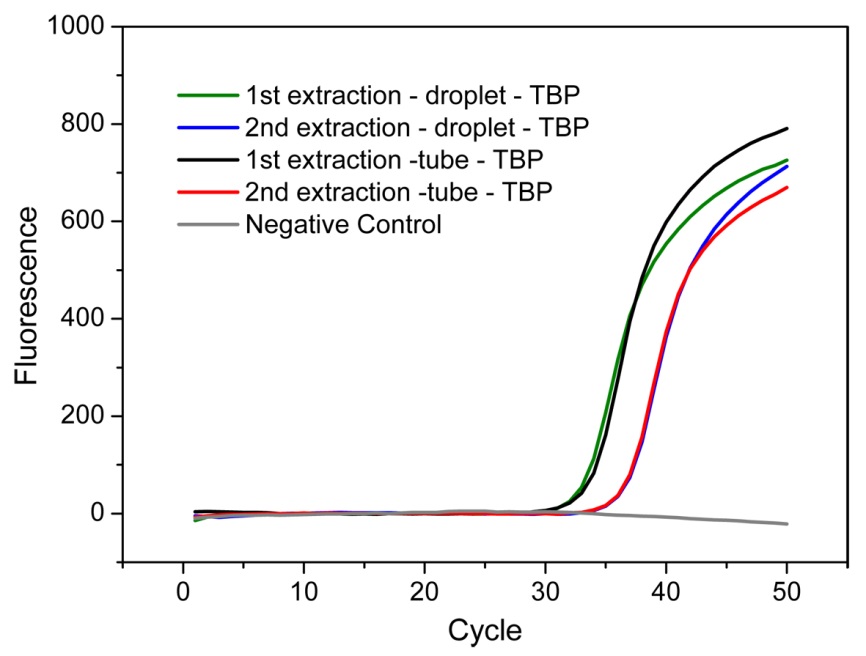


**Supplementary Figure N3:** qPCR curves for capture efficiency evaluation. qPCR curves obtained from the first and the second extraction of mRNA starting from an initial solution containing 1 ng of total RNA. The protocol was applied to TBP amplification, following the same protocol by the droplet microfluidic platform and conventional tube strategies.

**Supplementary Table N1:** Averages of triplicate experiments of the Ct values evaluated from the first Ct_1_ and the second Ct_2_ mRNA extraction by magnetic beads, in the droplet platform and with conventional tubes. The difference ∆Ct=Ct_2_-Ct_1_≈4 is similar for both conditions within experimental uncertainty.

|  | Ct_1_ | | Ct_2_ | | ∆Ct | |
| --- | --- | --- | --- | --- | --- | --- |
| droplet | | 32,43 ± 0,37 | | 36,59 ± 0,79 | | 4,16 |
| tube | | 32,23 ± 0,24 | | 35,95 ± 0,77 | | 3,72 |

The capture efficiency can be quantified by the following simple model: we quote Q_0_ the mRNA quantity present at the beginning in the droplet (typically corresponding to 2-3% of the 1ng of total RNA), and Q_ext_ and Q_res_, respectively, the quantities of mRNA extracted by the magnetic beads and left in the original solution during the first round. The fraction of mRNA captured on the beads is defined as$x$, i.e. the capture efficiency. Assuming that the beads are not saturated in mRNA, so that the capture efficiency x does not depend on the mRNA concentration in the range used here, one gets after the first mRNA capture and extraction:

$Q_{ext,1}=Q_{0}\cdot x$ $Q_{res,1}=Q_{0}-Q_{ext,1}=Q_{0}-Q_{0}\cdot x;$ (S1,S2)

and after the second mRNA extraction:

$Q_{ext,2}=Q_{res,1}\cdot x=(Q_{0}-Q_{0}\cdot x)\cdot x$ $Q_{res,2}=Q_{res,1}-Q_{ext,2};$ (S3,S4)

The Ct values are related to the mRNA quantity released in a given drop according to the equation:

$Ct=-3.3\cdot\log_{10}Q+A,$ (S5)

where A is a constant that depend on the expression level of the specific gene and 3.3 is the value for a the ideal PCR amplification efficiency. Defining$\Delta Ct=Ct_{2}-Ct_{1}$, one gets:

$\Delta Ct=-3.3\cdot\log_{10}(Q_{ext,2})-(-3.3\cdot\log_{10}(Q_{ext,1})) \to x=1-10^{-\frac{\Delta Ct}{3.3}}$ (S6)

the measured value ∆Ct=4±0.5, yields a capture efficiency$x$of (94±2)%. This capture efficiency is very high, and justifies a posteriori our initial hypothesis of non-saturation of the beads.

**Supplementary Note 3:**

The purification efficiency of the method, (i.e. the ability to eliminate most of non-messenger RNA) was evaluated. Non messenger RNA in the cell is mainly constituted by ribosomal RNA and other non-coding RNA, such as micro-RNA. In fact, mRNA represents only the 1-3% of the total RNA. Therefore, the purification efficiency is firstly represented in the reduction of the total RNA content in a given sample volume. This was evaluated using the microfluidic electrophoresis Bionalyzer Instrument (by Agilent Technology). Following the same protocol used for gene expression quantification described in the main text, mRNA was extracted by the magnetic tweezers from an initial droplet containing total RNA. The droplet containing the extracted mRNA and the effluent “waste” droplet containing the supernatant from the sample droplet after extraction were collected in separate tubes pre-filled with 2.5μL of water. The two solutions were analyzed in the Bioanalyzer. Typical results are reported in Supplementary Fig. N4: the two electropherograms display the same two major peaks, which we attribute to ribosomal RNA. Since the difference between total RNA contents mainly reflects the elimination of non-messenger RNA, we evaluated their ratio, which provides a lower bound higher than 100X for the purification efficiency (rejection factor of non-messenger RNA).


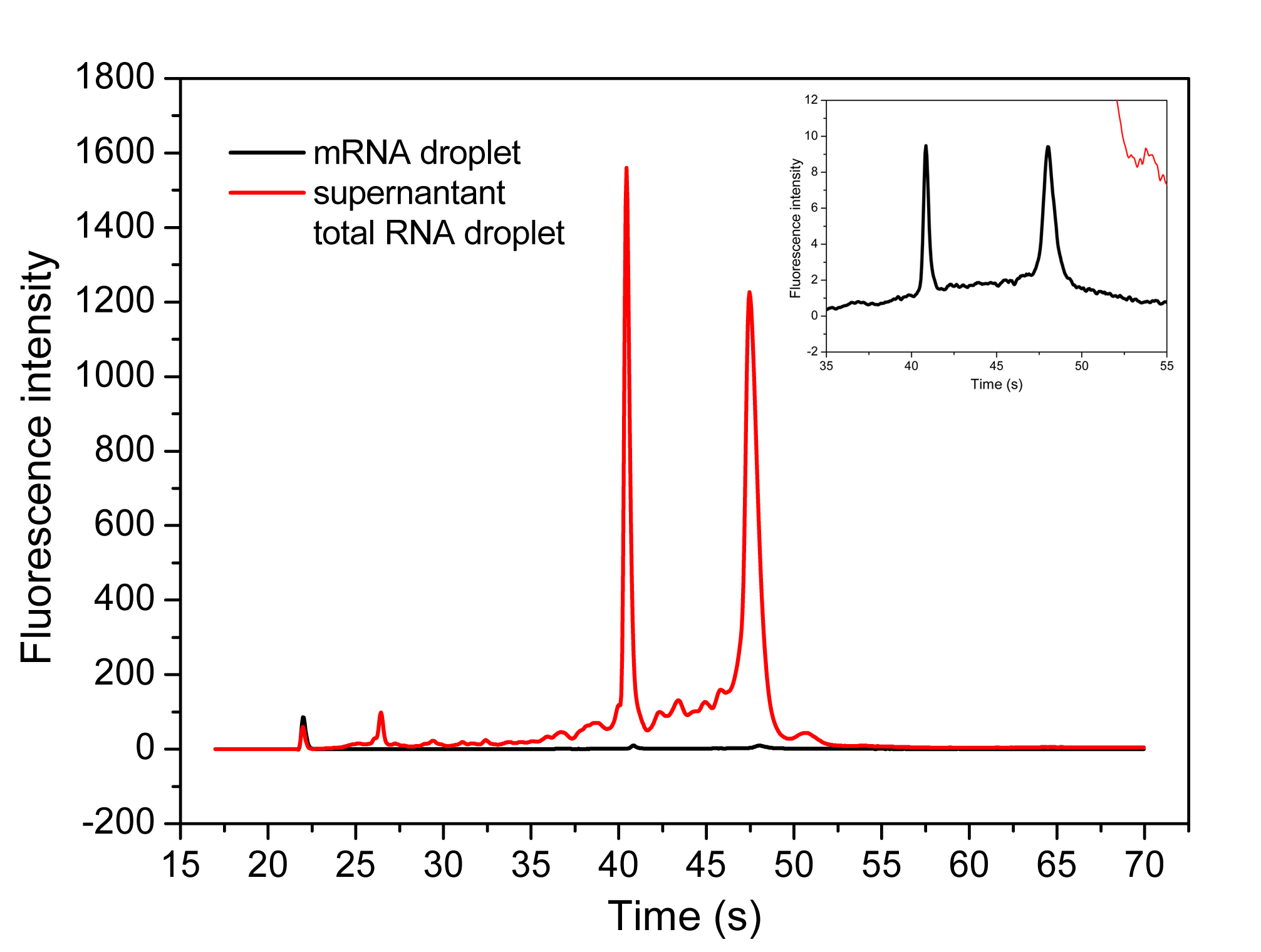


**Supplementary Figure N4:** evaluation of purification efficiency. Profiles obtained by the Bioanalyzer Instruments after the mRNA extraction from 25ng/droplet of total RNA solution (Universal Human Reference RNA, by Agilent Technologies): in black the result from the purified droplet (with a zoom in the upright inset), in red that from the supernatant. The electropherogram is typical for high quality total RNA from human cell lines, showing two major peaks corresponding to ribosomal RNA 18S and 28S (1.9 and 5 kb, respectively) (see e.g. Fig. 2 in ^1^). The concentration of the dominant ribosomal RNA is reduced by a factor >100 in the purified droplet (black trace).

**Supplementary Figure 1:**


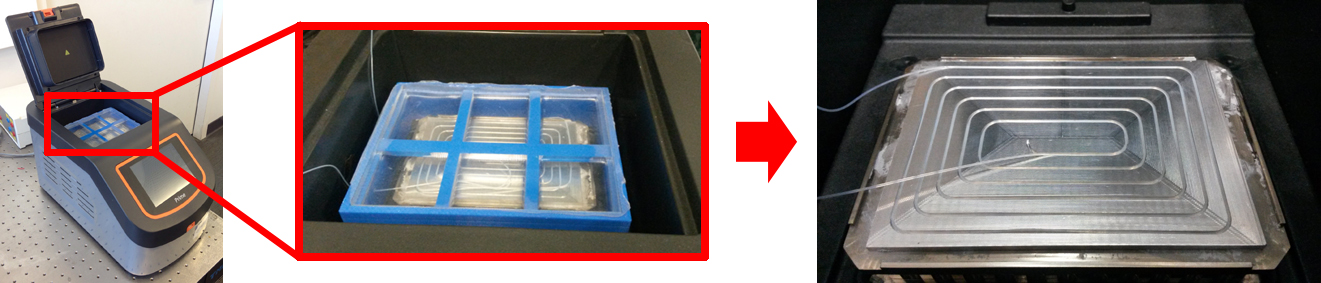


**Thermocycler for RT and PCR in droplet.** In order to perform the RT and PCR cycles on droplets, we adapted a commercial thermocycler (Techne Prime) equipped with a flat plate for glass slides (Techne in situ Hybridisation Adapter). As shown in the figure, an aluminum part with a groove for capillary holding is placed on the thermocycler plate and equipped with the PTFE capillary. The plate was prepared by a micro-milling machine ( Minitech Machinery Corporation, Georgia), using CATIA V5 as CAD/CAM software. A transparent PDMS box was placed over the plate in order to reduce heat convection and improve temperature stability, while keeping the observation of droplets possible. In order to optimize the thermo cycling, taking into account thermal conduction effects across the capillary holder, different thermocouples (250μm diameter, IT-24P, by PHYMEP) were placed in different spots of the holder plate and the thermocycler temperatures were adjusted by trial and error. In order to obtain 50±0.1°C, 60±0.1°C and 95±0.1°C in the capillary, the thermocycler temperature had to be fixed at 50.8°C, 60.5°C and 94.3°C, respectively.

**Supplementary Table 1:**

**Influence of the enzyme quantity for RT-PCR in droplet.** Supplementary Table 1 shows the Ct values obtained from total RNA samples (Universal Human Reference RNA, by Agilent Technologies) at a concentration of 1 ng/droplet (4ng/μL), for expression level of Actinβ, a “housekeeping” gene known for showing a high level of expression. A commercial standard and a highly expressed gene were chosen to avoid risks of sample to sample variability or RNA degradation and to focus on the characterization of the enzyme quantity. The same protocol as for testing RT-PCR in droplets was used, i.e. RT (5 minutes at 50°C) and then 15 cycles of PCR (30 sec at 95°C; 30 sec at 60°C). The enzyme concentration in the RT-PCR mix droplet was varied between 2 to 300% of the recommended concentration for in tube format. Each reported Ct is the result of the average of three experiments, and the relative standard deviation is reported as error. The same Cts were observed, except for 2% of recommended concentration (condition 5). These results suggest that the recommended enzyme concentration in tubes (condition 1) can also be used in droplet, being on a plateau of optimal performances with comfortable margins on both sides.

|  | Enzyme quantity | Ct |
| --- | --- | --- |
| Condition 1 | 100% | 12,13±0,22 |
| Condition 2 | 300% | 12,17±0,15 |
| Condition 3 | 50% | 12,14±0,21 |
| Condition 4 | 20% | 12,17±0,11 |
| Condition 5 | 2% | 26,98±0,15 |

**Supplementary Figure 2:**


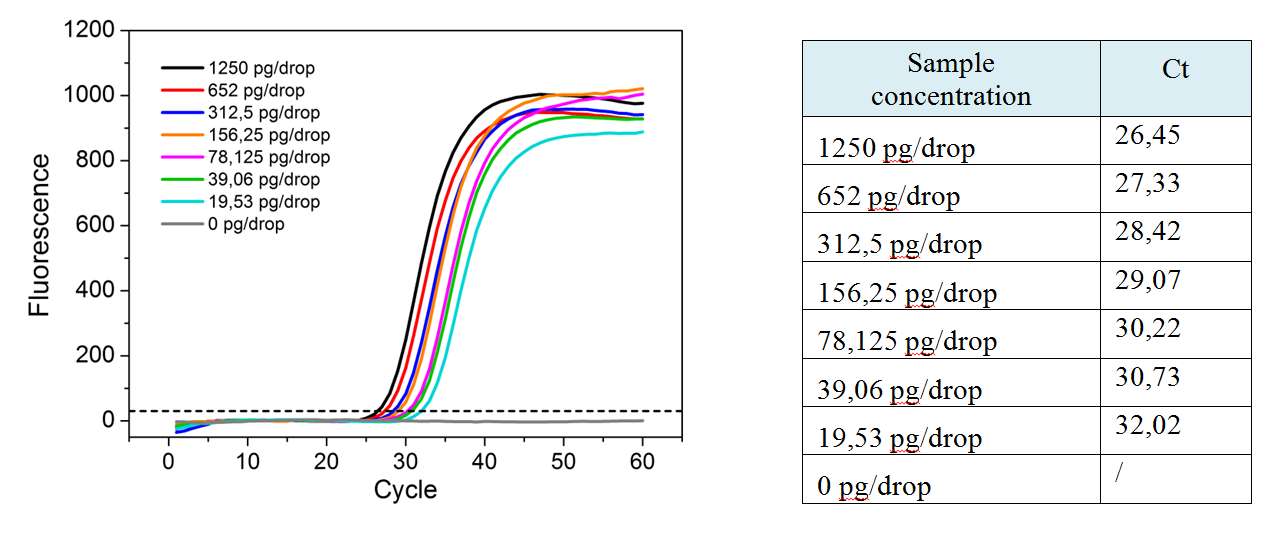


**Example of qPCR curves and relative Ct values.** The qPCR curves obtained for the determination of HER2 expression based on various quantities of total RNA from SKBR3 cell line are illustrated on the left. The horizontal dashed line defines the threshold for the determination of the Ct values reported in the table on the right. Three similar series of experiments were performed and averaged to yield the calibration curves reported in Fig. 3b of the main text.

**Supplementary Table 2:**

**Ct values of patient samples obtained by the Hôpital Saint Louis (clinical diagnosis department) and by the droplet microfluidic platform.** The first column contains the anonymization patient codes. The blue area contains the hospital results and the yellow one those of the microfluidic platform. Each area is divided in different columns showing the Ct values for TBP (TBP Ct) and HER2 (HER2 Ct), the ΔCt of the sample (HER2 ΔCt), theΔΔCt normalization with the reference sample and the *Fd* values. Immunohistochemistry diagnostic results (HER2 IHC) obtained at the Hospital Saint Louis are also reported as confirmation. In both areas, positive HER2+ samples are marked in red.

**
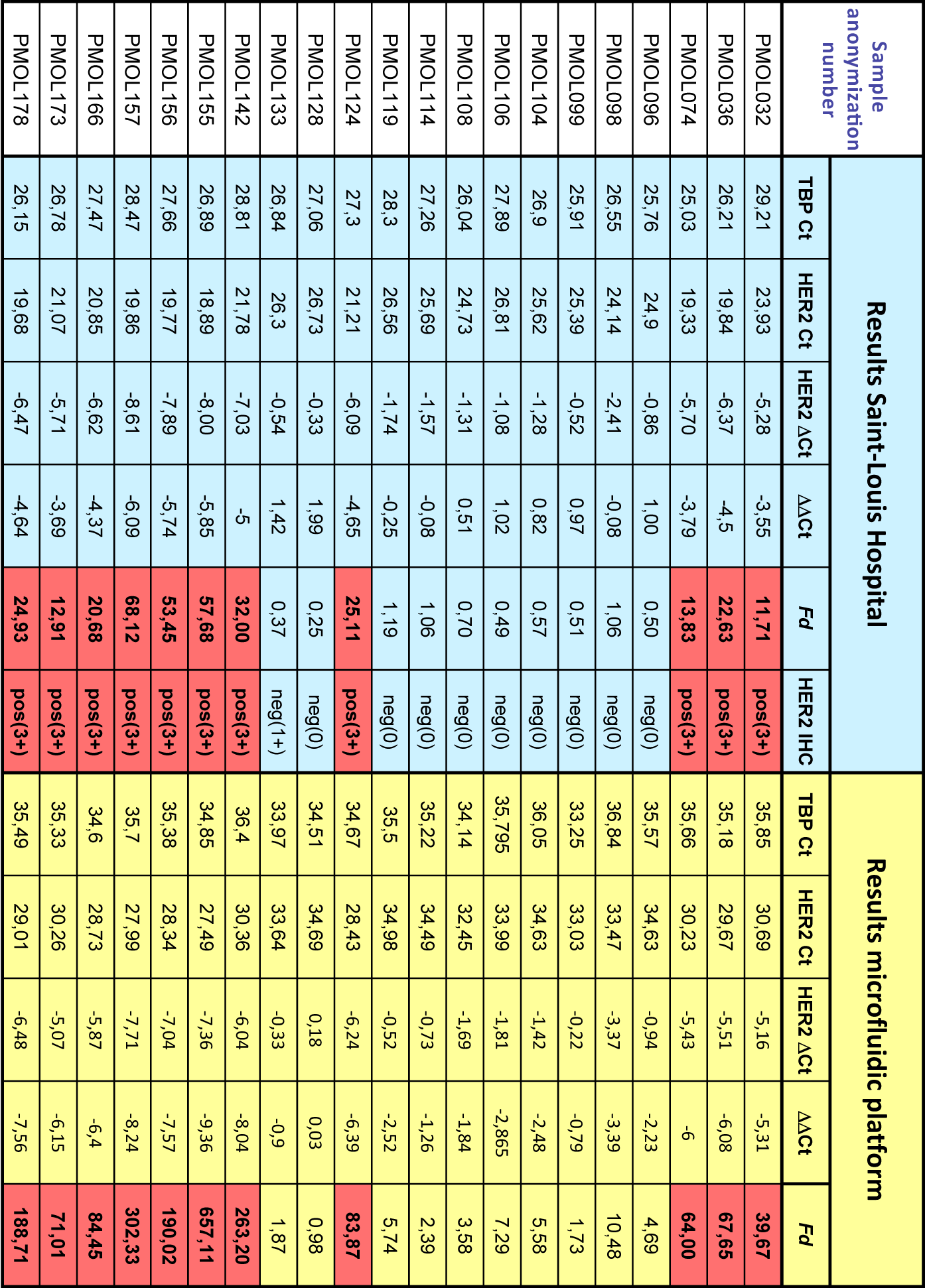
**

**Supplementary Figure 3:**

**
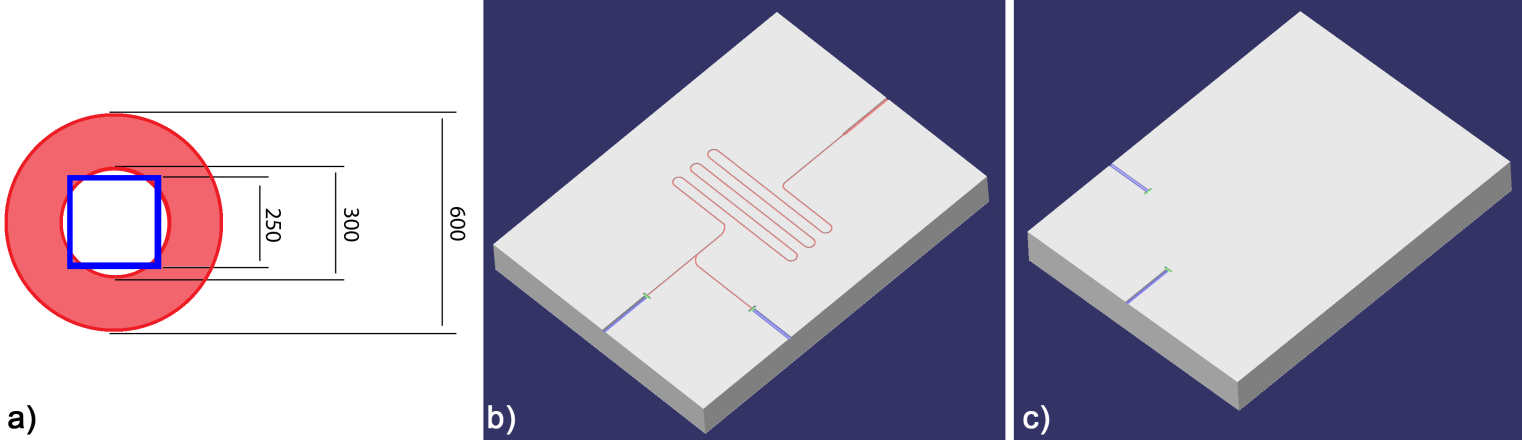
**

**Inlets /outlets microfabrication scheme.** a) Scheme of the side view of the microfluidic PDMS channel (blue square) and the aligned PTFE capillary (red circles): the quotes are in micrometers and represent the real design. The inlets and outlets are aligned with the microfluidic channels in order to avoid any possible droplet break-up during the entrance and the exit of the devices. From the microfabrication point of view, this is obtained by preparing one mold for each side of the device: one with the channels and a space for holding the capillary (panel b) and the other containing just the capillary holders (panel c). Binding the two parts in alignments allows an automated collinear centering of the axes of the capillaries and channel.

**Reference - Supporting Information**

1. Krupp, G.*, Stringent RNA quality control using the Agilent 2100 bioanalyz*er. (2005) Available at: https://www.agilent.com/cs/library/applications/5989-1086EN.pdf (Accessed: 14th March 2016)
